# Full-genome analysis of emerging Coxsackievirus B4 genotype D strains associated with herpangina in eastern China

**DOI:** 10.1099/mgen.0.001656

**Published:** 2026-02-26

**Authors:** Xiaosong Hu, Yuxiang Sun, Bo Xing, Linjie Hu, Zhenzhen Liang, Jian Fu, Yuxia Liang, Yijuan Chen, You Li, Lingxia Chen, Lijun Wang, Weiting Wang, Shenyu Wang, Yihan Lu

**Affiliations:** 1 Department of Immunization Program, Zhejiang Provincial Centre for Disease Control and Prevention, Hangzhou, PR China; 2 Department of Epidemiology, Ministry of Education Key Laboratory of Public Health Safety, School of Public Health, Fudan University, Shanghai, PR China; 3 Institute of Medical Research, Huashan Hospital, Fudan University, Shanghai, PR China; 4 Department of Prevention and Control of Infectious Diseases, Zhejiang Provincial Centre for Disease Control and Prevention, Hangzhou, PR China; 5 Zhejiang Key Lab of Vaccine, Infectious Disease Prevention and Control, Zhejiang Provincial Centre for Disease Control and Prevention, Hangzhou, PR China; 6 Department of Epidemiology, Centre for Global Health, School of Public Health, Nanjing Medical University, Nanjing, PR China; 7 Shanghai Institute of Infectious Disease and Biosecurity, Fudan University, Shanghai, PR China

**Keywords:** enterovirus, Coxsackievirus B4, cross-border transmission, herpangina, recombination, phylogenetic analysis

## Abstract

In 2024 herpangina surveillance in Yuhuan City, China, two Coxsackievirus B4 strains (Z168, Z296) were identified. Full-length genome sequencing revealed that both strains belonged to genotype D that was first documented in eastern China. The two strains showed the highest nucleotide similarity to Coxsackievirus B4 strains isolated in the UK (2017). In the recombination events, both major parental strains were identified as Coxsackievirus B4 strains isolated in France (2015 and 2017). Echovirus 11 and Coxsackievirus B5 were detected as minor parental strains, indicating intertypic recombination. Phylogenetically, Z168 and Z296 clustered closely with European genotype D strains (2013–2024) but were distant from previously reported Chinese genotype D strains (Yunnan 2013–2016; Tianjin 2019). Bayesian phylogeographic reconstruction suggested a complex global transmission history of Coxsackievirus B4 with increasing cross-border transmission in recent decades, including genotypes A, D and E transmitting among North America, Europe and Asia. Both strains possessed a unique amino acid substitution within a putative neutralizing epitope, suggesting potential antigenic evolution. These findings indicate that the Z168 and Z296 strains likely represent a recent European introduction, forming a lineage distinct from earlier Chinese genotype D isolates. This highlights the need for molecular surveillance to track Coxsackievirus B4 emergence and evolution.

**Impact Statement**

This study identifies two emerging Coxsackievirus B4 (CVB4) genotype D strains through sentinel herpangina surveillance in eastern China, representing a previously unrecognized lineage distinct from earlier Chinese isolates. Using full-genome sequencing, recombination analysis and Bayesian phylogeography, we show that these viruses likely originated from recent European introductions and exhibit intertypic recombination as well as a unique amino acid substitution within a putative neutralizing epitope. Our findings provide strong genomic evidence of ongoing cross-border transmission of CVB4 and highlight increasing genetic complexity driven by recombination and adaptive evolution. This study updates the global evolutionary framework of CVB4, challenges the long-held assumption of geographically isolated genotype circulation and emphasizes the urgent need for enhanced molecular surveillance to detect emerging lineages with potential public health significance.

## Data Summary

The two strains Z168 and Z296 were deposited under the accession number PV453396 and PV453397, respectively, in the GenBank. The CVB4 and other sequences used in this study are available in the GenBank of the National Center for Biotechnological Information (https://www.ncbi.nlm.nih.gov/genbank/). The accession numbers of all sequences are listed in the Tables S1–S6.

## Introduction

Coxsackieviruses are non-enveloped viruses with a positive-sense, linear ssRNA genome ~7500 nt in length [1]. Group B Coxsackieviruses (CVBs), comprising six recognized serotypes (CVB1–CVB6), are classified within the species Enterovirus B, genus *Enterovirus*, family *Picornaviridae*, in accordance with the latest nomenclature guidelines from the International Committee on Taxonomy of Viruses [2]. CVBs have been implicated in a broad spectrum of clinical conditions, including gastrointestinal disorders; myocarditis; pneumonia; aseptic meningitis; encephalitis; herpangina; hand, foot and mouth disease (HFMD); and hepatitis [3,4].

Among the CVBs, CVB4 is a globally circulated serotype with high virulence [3,5]. It has been isolated from patients presenting with various diseases worldwide, such as HFMD/herpangina in China (2016) [6], aseptic meningitis in Brazil (2017) [7] and pneumonia cases in China (2018) [8]. Notably, a large HFMD outbreak in Shandong Province, China, from 2010 to 2011 was predominantly caused by CVB4, underscoring its epidemic potential [9]. Furthermore, CVB4 has been increasingly recognized for its potential role in the pathogenesis of type 1 diabetes through multiple mechanisms [10,12]. Generally, CVB4 exhibits significantly higher mortality rates than other enterovirus serotypes [5].

Genetically, CVB4 has been classified into five genotypes (A–E), based on nucleotide divergence within the VP1 region [9]. Historically, genotype D has predominated in CVB4 outbreaks outside China, while genotype E has been commonly prevalent in Chinese epidemics [13]. However, a study investigating CVB4 strains isolated from HFMD patients in Yunnan Province, China (2013–2016), revealed that most strains belonged to genotype D and a minority belonged to genotype A, which marked the first identification of these two genotypes in China [14]. Additionally, CVB4 is characterized by considerable genetic variability. Previous studies have reported a high frequency of recombination and genetic variability in CVB4, suggesting potential shifts in its pathogenicity, virulence and antigenicity [13,15, 16]. In 2019, a recombinant CVB4 genotype D strain was isolated from an HFMD patient in Tianjin Municipality, China [17].

Herpangina is a common acute upper respiratory tract infection primarily affecting preschool-aged children under 6 years old, and it shares a similar pathogen spectrum with HFMD [18,19]. In 2024, we conducted a herpangina sentinel surveillance in Yuhuan City, Zhejiang Province, China, and identified two CVB4-positive cases. Full-length genome sequencing revealed that both belonged to genotype D – a genotype distinct from the CVB4 strains previously reported to be circulating in Zhejiang [20]. This study aims to characterize these two CVB4 strains, providing data and evidence that may inform future disease surveillance strategies and public health interventions.

## Methods

### CVB4 cases

In 2024, we conducted sentinel surveillance of herpangina outbreaks at two hospitals in Yuhuan City, Zhejiang Province of eastern China. A total of 133 clinical cases were enrolled, of which clinical data and biological specimens were collected. Among these cases, two cases tested positive for CVB4 (as described in ‘Sample collection, virus serotyping and full-length sequencing’); other cases tested positive for CVA2 and CVA5 (unpublished data).

Case 1: Patient Z168 was a 4-year-old boy who developed symptoms on 27 May 2024. He was diagnosed with herpangina on 29 May 2024 at Yuhuan People’s Hospital. His main clinical manifestations included fever, with a peak temperature of 39.0 °C, and vesicular ulcerations localized to the oral and pharyngeal mucosa.

Case 2: Patient Z296 was a 4-year-old girl who developed symptoms on 16 June 2024 and was diagnosed with herpangina on 19 June 2024 at Yuhuan Second People’s Hospital. Her symptoms included fever, with a maximum temperature of 38.2 °C, accompanied by vesicular ulcers in the oral and pharyngeal regions.

These two cases reported no social connection, and their residences were 15 km apart.

### Sample collection, virus serotyping and full-length sequencing

In the sentinel surveillance, nasopharyngeal swab specimens were collected from herpangina cases reported by sentinel hospitals on the day of case notification. All specimens were transported at 4 °C and subsequently stored at −80 °C until further processing. Viral nucleic acids were extracted from nasopharyngeal swabs using the BioGerm Viral Nucleic Acid Extraction Kit (BioGerm, Shanghai), operated on the BG-Flex-48 Automated Nucleic Acid Extraction System (BioGerm). The presence and quality of enterovirus nucleic acids were assessed using the BioGerm Enterovirus qPCR Detection Kit. Samples yielding a cycle threshold (CT) value below 30 were considered positive for enterovirus RNA.

Positive samples were further subjected to serotyping via PCR targeting the 5′ UTR, using the Enterovirus Genotyping PCR Kit (BioGerm). The reactions were carried out with serotype-specific primers (Table S11, available in the online Supplementary Material) under the following thermal conditions: reverse transcription at 50 °C for 10 min and initial denaturation at 95 °C for 5 min, followed by 45 cycles of denaturation at 95 °C for 10 s and combined annealing/extension at 55 °C for 40 s, with fluorescence acquisition at the end of each cycle. A 2 µl aliquot of each PCR product was analysed by 2% agarose gel electrophoresis to confirm the presence of target-sized amplicons. Verified PCR products were sequenced using the Sanger method with the ABI 3730xl Genetic Analyzer and BigDye® Terminator v3.1 Cycle Sequencing Kit. Resulting sequences were assembled using SeqMan software (DNASTAR) and serotyped by alignment against the NCBI blast database (https://blast.ncbi.nlm.nih.gov/Blast.cgi) and the Enterovirus Genotyping Tool (https://www.genomedetective.com/app/typingtool/etv/).

In the above process, two cases (Z168 and Z296) were identified testing positive for CVB4. Their samples showed the CT values of ≤25 that were applicable for full-length sequencing. For full-length genome sequencing, reverse transcription polymerase chain reaction was then performed using overlapping primer pairs covering the entire CVB4 genome (Table S12). The thermal conditions were as follows: 53 °C for 32 min and 95 °C for 5 min, followed by 40 cycles of 95 °C for 30 s, 52 °C for 30 s and 70 °C for 1 min 30 s, with a final extension at 72 °C for 5 min and a hold at 4 °C. These two samples underwent viral RNA enrichment using the Tarich Enterovirus A Nucleic Acid Enrichment Kit SJ-CX-601 (BioGerm), followed by library preparation with the Whole Genome DNA Library Preparation Kit SJ-CX-606 (BioGerm). Sequencing libraries were then processed on the DNBSEQ-T7 platform (MGI Tech Co., Ltd., Shenzhen, China) using a 150 bp paired-end (PE150) strategy. Each sample generated a minimum of 1 Gb of high-quality data, with Q30 scores exceeding 85%.

Following the full-length sequencing, raw reads were subjected to quality assessment using FastQC (v0.11.9). Adapter sequences and low-quality reads were trimmed using Fastp (v0.23.2). Clean reads were aligned to reference enterovirus genomes using BWA (v0.7.17) and SAMtools (v1.16.1). Consensus full-length sequences were subsequently generated using Ivar (v1.3.1). The two strains Z168 and Z296 were deposited under the accession number PV453396 and PV453397, respectively, in the GenBank.

### CVB4 sequence dataset construction

All near-complete CVB4 genome sequences (ranging from 6,000 to 7,600 nt in length) available in the GenBank database were retrieved as of 1 March 2025. A total of 110 complete coding sequences (CDSs) of CVB4 (108 from the GenBank and 2 from this study) were included (dataset CVB4 CDS; Table S1). These sequences were aligned using mafft v7.525 with the FFT-NS-i algorithm for subsequent nucleotide and amino acid similarity analyses [21]. In addition, prototype strains of other CVB-like enteroviruses, as described elsewhere [22], were retrieved from the GenBank and constituted another sequence dataset in combination with 17 reference CVB4 sequences (dataset CVB4+CVB-like CDS; Table S2). This dataset was used to compare nucleotide and amino acid similarities among different CVB-like enteroviruses.

Moreover, CVB4 VP1 sequences were compared. All CVB4 sequences exceeding 884 nt in length available in the GenBank were collected as of 1 March 2025. These sequences were aligned and trimmed using mega v11.0.13 [23]. Redundant or incomplete sequences were excluded, as were those lacking metadata on collection date or geographic location. Ultimately, a total of 352 VP1 sequences were included (dataset CVB4 VP1; Table S3) for phylogenetic analysis. Of these, a total of 199 reference sequences were selected for further investigation of spatial and temporal dynamics, based on diversity in sampling time, geographic location and nucleotide similarity (dataset CVB4 reference VP1; Table S4).

Lastly, the CDS and nucleotide sequences corresponding to the P1, P2 and P3 coding regions of strains Z168 and Z296 were subjected to blast analysis to identify sequences in the GenBank with high similarity. The most closely related sequences (based on blast analysis results ranked by per cent identity and with complete P1/P2/P3 regions) were extracted into two separate datasets (datasets Z168 blast CDS and Z296 blast CDS; Tables S5 and S6), which were used for subsequent recombination analyses of Z168 and Z296, respectively.

### Nucleotide and amino acid similarity calculation

A neighbour-joining (NJ) phylogenetic tree was reconstructed in mega v11.0.13 using the dataset of CVB4 VP1 sequences. Nucleotide and amino acid identities were calculated among the two CVB4 strains isolated in this study, CVB-like prototype strains and reference CVB4 strains retrieved from the GenBank using the Clustal Omega Multiple Sequence Alignment tool available on the EBI web server (https://www.ebi.ac.uk/jdispatcher/msa/clustalo) [24]. Heatmaps were illustrated using the R pheatmap package [25]. SimPlot software v3.5.1 was employed to perform similarity analysis across the complete CDS of the two CVB4 strains [26].

### Detection of recombination events

Recombination analysis was initially conducted using blast analysis. Moreover, the Recombination Detection Program v4.46 (RDP4) [27] was employed that integrates seven different algorithms – RDP, GENECONV, Chimaera, MaxChi, Bootscan, SiScan and 3Seq – for comprehensive identification of potential recombination events. Those events supported by at least four of the seven methods were considered credible and retained for further investigation. To validate the recombination signals, bootscan analysis was performed using SimPlot software v3.5.1. The NJ trees were generated using a sliding window of 200 nt with a step size of 20 nt, applying the Kimura 2-parameter substitution model and 1,000 bootstrap replicates to assess branch support.

### Analysis of spatial and temporal dynamics

Reference CVB4 sequences were selected from the dataset CVB4 VP1 to explore the spatial and temporal dynamics of CVB4 evolution. To assess the temporal signal in the dataset, root-to-tip regression analysis was conducted using TreeTime v0.11.4 [28]. The optimal nucleotide substitution model was determined using the ModelFinder module in IQ-TREE v2.4.0 [29], with the GTR+F+R4 model selected based on the Bayesian information criterion score.

Bayesian phylogenetic analysis was carried out using BEAST v1.10.4 [30] to reconstruct a maximum clade credibility (MCC) tree via the Markov Chain Monte Carlo (MCMC) method. Model comparison was performed using path sampling and stepping-stone sampling approaches to evaluate combinations of clock models and tree priors based on marginal likelihood estimation (Table S7). The uncorrelated exponential relaxed clock model and the expansion coalescent tree prior were ultimately selected as the best-fitting combination. The MCMC analysis was run for 2 billion generations with sampling every 2,000 steps. Country of origin was incorporated as a discrete trait to infer spatiotemporal transmission patterns. Convergence of model parameters was assessed using Tracer v1.7.2, with effective sample size values greater than 200 considered to be indicative of sufficient convergence. The MCC tree was generated using TreeAnnotator v1.10.4, discarding the first 10% of sampled trees as burn-in. Tree visualization was conducted using TVBOT (https://www.chiplot.online/tvbot.html) [31].

In addition, the BaTS (Bayesian Tip-Significance Testing) tool v1.0 [32] was employed to evaluate the association between phylogenetic clustering and geographic location. Statistical support for geographic structure was quantified using the association index (AI), parsimony score and maximum monophyletic clade metrics. To reconstruct the spatial transmission patterns of CVB4, Bayesian Stochastic Search Variable Selection (BSSVS) analysis was conducted using an asymmetric substitution model in BEAST v1.10.4. Transmission routes were assessed using Bayes factor (BF) and posterior probability (PP) values. Links with BF ≥3 and PP >0.5 were regarded as significantly supported. The spatial transmission routes were then visualized using Adobe Illustrator v29.5.1 on a standard world map [map approval no. GS(2016)1663].

### Analysis of amino acid substitution

CVB4 VP1 amino acid consensus sequences were generated using MegAlign from the Lasergene v7.0 package [33], based on the CVB4 strains isolated in China and belonging to genotypes A, D and E. These consensus sequences were then compared to the VP1 sequences of strains Z168 and Z296 to identify amino acid substitutions. Protein structure modelling of the VP1 of strains Z168 and Z296 was performed using the I-TASSER web server [34]. The resulting 3D models were visualized using ChimeraX v1.8 [35] to illustrate the spatial distribution of the amino acid substitutions.

## Results

### Nucleotide and amino acid similarity of two CVB4 strains

**Fig. 1.**
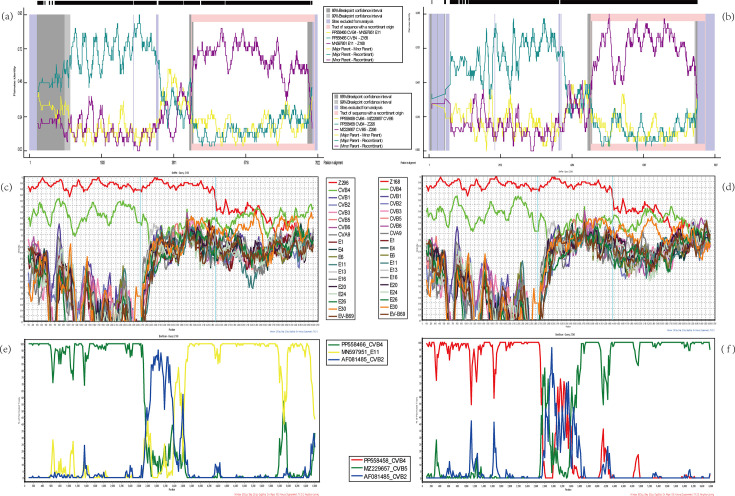
(**a**) Recombination detection plot of Z168 by using RDP4. (**b**) Recombination detection plot of Z296 by using RDP4. (**c**) Simplot of general recombination events identified in P2 and P3 regions of the Coxsackievirus B4 full-length sequence Z168 isolated in the study, as compared to dataset CVB4+CVB-like CDS, by using SimPlot. (**d**) Simplot of general recombination events identified in the P2 and P3 regions of the Coxsackievirus B4 full-length sequence Z296 isolated in the study, as compared to dataset CVB4+CVB-like CDS, by using SimPlot. (**e**) Bootscan plot of Z168 and its inferred major and minor parental strains as determined by RDP4 analysis. (**f**) Bootscan plot of Z296 and its inferred major and minor parental strains as determined by RDP4 analysis.

The two CVB4 strains isolated in this study, Z168 and Z296, showed high similarity to each other, with nucleotide and amino acid identities of 93.04 and 98.26%, respectively (Table 1). Among the three CDS regions, the P1 and P2 demonstrated consistently high similarity between the two strains, with nucleotide and amino acid identities of ~97 and 99%, respectively. In contrast, the P3 region showed lower similarity, with nucleotide and amino acid identities of 85.59 and 96.56%, respectively. Similarly, SimPlot analysis yielded consistent findings (Fig. 1), revealing that Z168 and Z296 shared over 95% nucleotide similarity across the P1 and P2 regions; however, in the P3 region, the nucleotide similarity markedly decreased to below 90% and even dropped to less than 85% in the latter portion of P3.

**Table 1.** Amino acid and nucleotide similarity between Z168, Z296 and the reference strains in the dataset CVB4 CDS

	Z168-CVB4 CDS dataset		Z296-CVB4 CDS dataset		Z168-Z296	
	Nucleotide (%)	Amino acid (%)	Nucleotide (%)	Amino acid (%)	Nucleotide (%)	Amino acid (%)
Complete CDS	79.76–86.84	96.01–97.80	79.70–86.05	96.52–97.62	93.04	98.26
P1	80.59–91.19	97.18–98.71	80.82–91.27	97.30–98.94	96.91	99.29
P2	78.43–83.88	94.43–97.39	78.78–84.23	95.12–98.08	97.1	98.95
P3	78.18–87.26	95.11–97.88	77.43–83.61	94.31–96.83	85.59	96.56

Moreover, the identities between Z168 and other sequences in the dataset CVB4 CDS ranged from 79.76 to 86.84% (nucleotide) and 96.01 to 97.80% (amino acid). For Z296, the identities were 79.70–86.05% (nucleotide) and 96.52–97.62% (amino acid). The two strains showed the highest similarity to CVB4 strains isolated in the UK (GenBank accession number MG451808, 2017). Compared with the P2 and P3 regions, the P1 region exhibited higher conservation when aligned with other CVB4 CDSs.

**Fig. 2.**
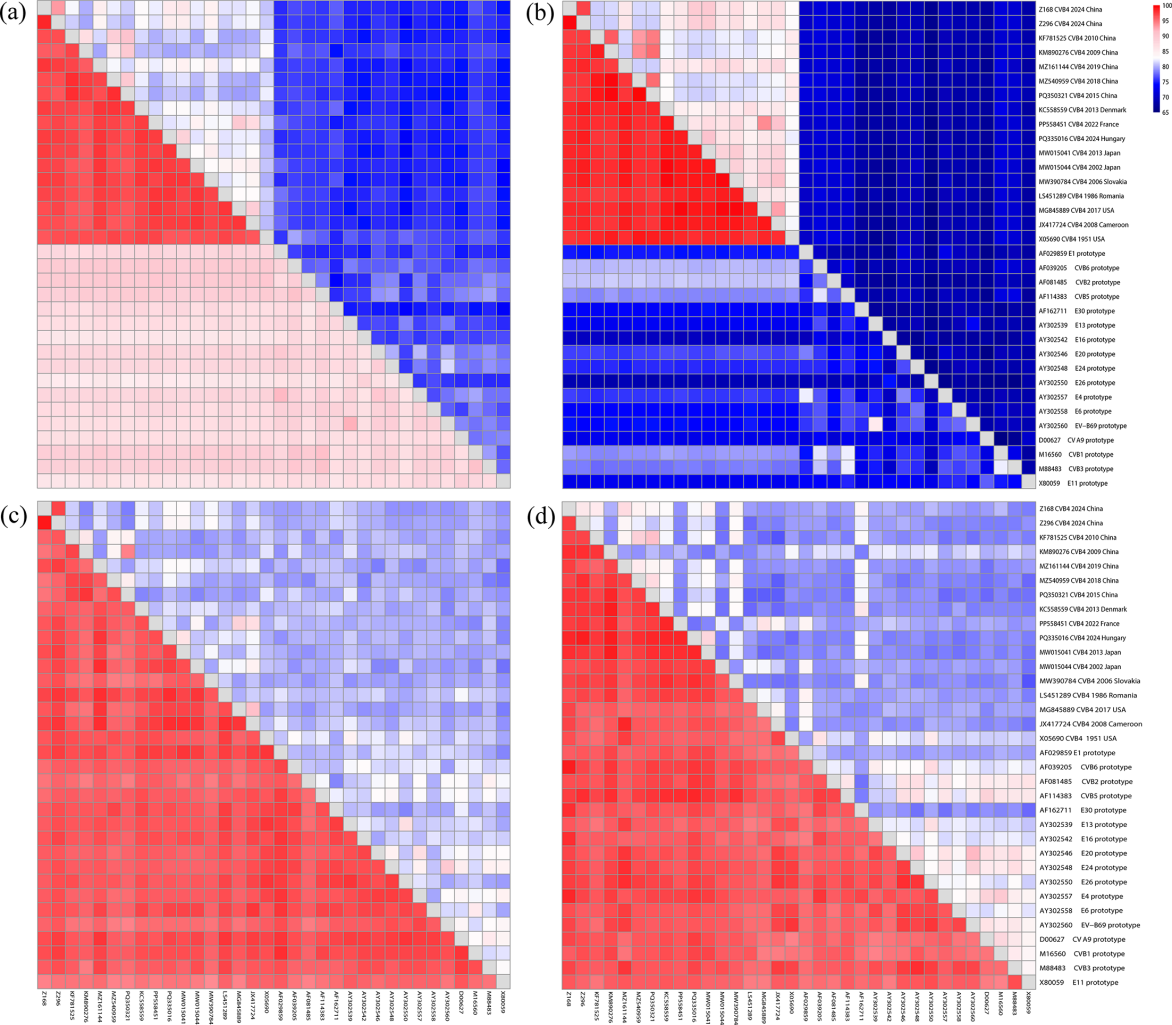
Heatmap of pairwise nucleotide and amino acid similarities for (**a**) complete CDSs of CVB4, (**b**) P1 region, (**c**) P2 region and (**d**) P3 region based on the CVB4+CVB-like CDS (Table S2). The upper right triangle shows nucleotide similarity, and the lower left triangle shows amino acid similarity.

A similarity heatmap was generated including several CVB-like enterovirus prototype strains and reference CVB4 strains in the dataset CVB4+CVB-like CDS (Fig. 2). The two strains in this study showed high similarity to CVB4 strains isolated in France (PP558451, 2022) and Hungary (PQ335016, 2024). Moreover, among the CVB-like enteroviruses, echovirus 30 prototype (E30; AF162711, 1958) displayed relatively high similarity to the P2 and P3 regions of Z168 and Z296, compared to other enteroviruses. Additionally, Z296 exhibited greater divergence in the P3 region compared to Z168, showing lower similarity to most sequences in the dataset.

### Recombination events

The sequences of Z168 and Z296 exhibited high similarity in the P1 and P2 regions but showed significant divergence in the P3 region, suggesting recombination events. Preliminary blast analysis identified potential recombinant strains based on the dataset Z168 blast CDS and Z296 blast CDS. Further analysis using RDP4 software revealed distinct recombination events for each strain. For Z168, recombination was detected between CVB4 and echovirus 11 (E11), with the major parental strain identified as CVB4 strain (PP558466, isolated in France, 2015) and the minor parental strain as E11 strain (MN597951, Guangdong Province, China, 2019). In contrast, Z296 was identified as a recombinant between CVB4 and CVB5, with CVB4 strain (PP558458, France, 2017) serving as the major parent and CVB5 strain (MZ229657, Hubei Province, China, 2019) as the minor parent (Table S8). These recombination events in the P3 region were further supported by bootscan analysis using SimPlot software (Fig. 1). Maximum-likelihood trees reconstructed from the full CDS, P1, P2 and P3 regions of the Z168 blast dataset (Table S5) and the Z296 blast dataset (Table S6), both comprising CVB4 sequences and their potential recombinant counterparts, are shown in Figs S1 and S2.

### Phylogenetic reconstruction and spatiotemporal pattern

**Fig. 3.**
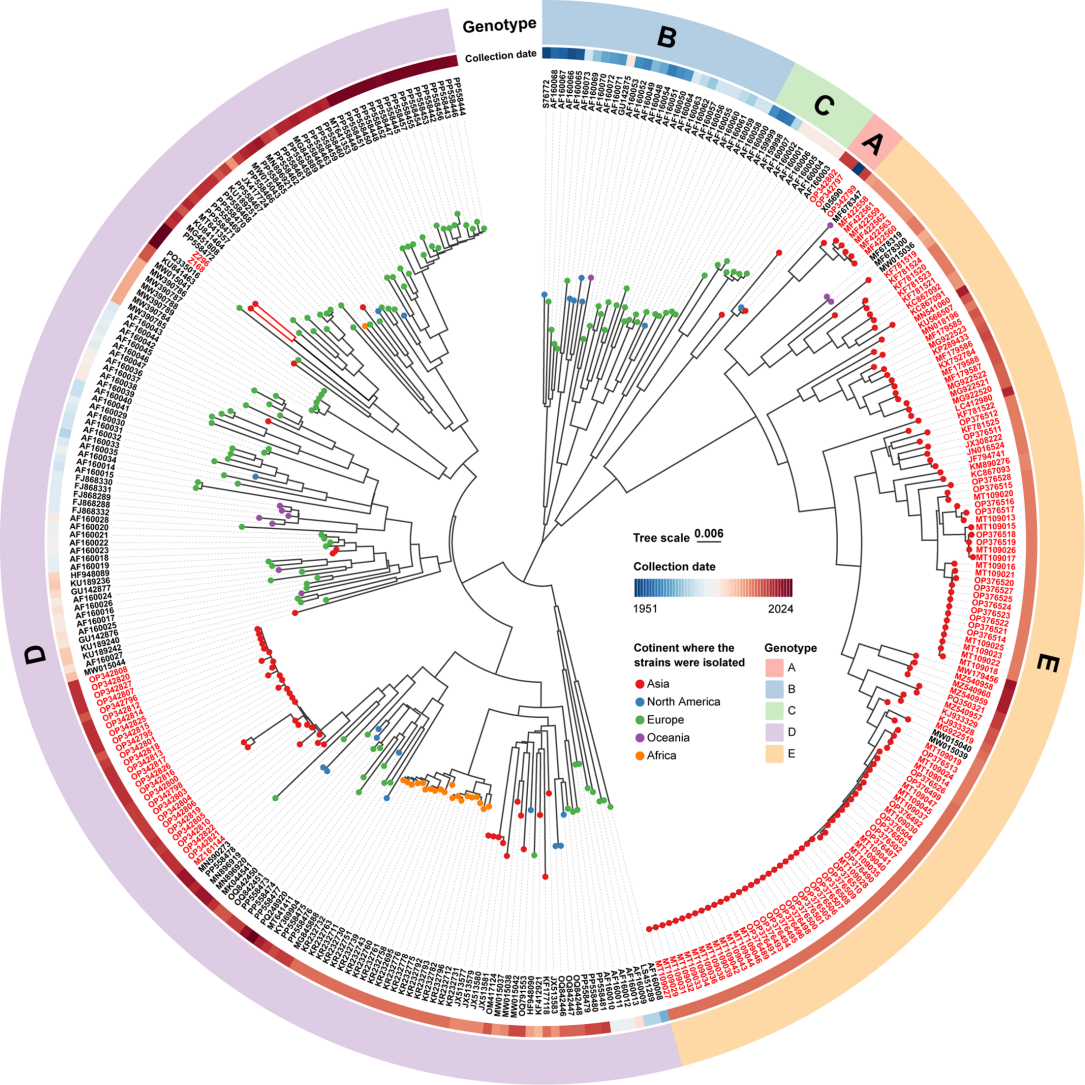
An NJ phylogenetic tree reconstructed based on the dataset CVB4 VP1 (Table S3). The circle is divided into four concentric layers to display the following information. The innermost layer uses coloured leaf labels to indicate the geographic origin of the strains. The second layer primarily shows strain labels, with those highlighted in red denoting isolates from China. The third layer displays the collection years of the strains. The outermost layer represents genotypes. The branches of the two strains isolated in this study are highlighted in red lines.

A phylogenetic tree was reconstructed using the NJ method for genotype classification based on the dataset CVB4 VP1 (Fig. 3). Generally, CVB4 strains isolated in China were classified to genotypes A, D and E. The two CVB4 strains isolated in this study, Z168 and Z296, clustered closely and were classified as genotype D. They showed close phylogenetic relationships with strains isolated in Hungary (PQ335016, 2024), the UK (MG451808 and MT641357, 2017), France (PP558471, 2015; PP558468, PP558469, PP558470 and PP558472, 2016) and Russia (KU841464, 2013). In contrast, other genotype D strains in China, including those isolated in Yunnan Province (*n*=26; 2013–2016) and Tianjin Municipality (MZ161144, 2019), were phylogenetically close to strains detected in the USA (MN896919 and MN896920, 2019), France (PP558478, 2017; and MN590273, 2019) and Turkey (MK044541, 2016). Therefore, genotype D strains consisted of two clusters in China. Additionally, genotype E has circulated in China for the longest period (2007–2019) and across the widest geographic range, making it the predominant genotype in the country. In Zhejiang Province, where Z168 and Z296 were isolated in our study, a genotype E strain (KP289433, 2013) had also been previously identified. Except for sporadic detections in Australia and Japan between 2006 and 2011, genotype E has only been identified in China, indicating strong regional specificity.

**Fig. 4.**
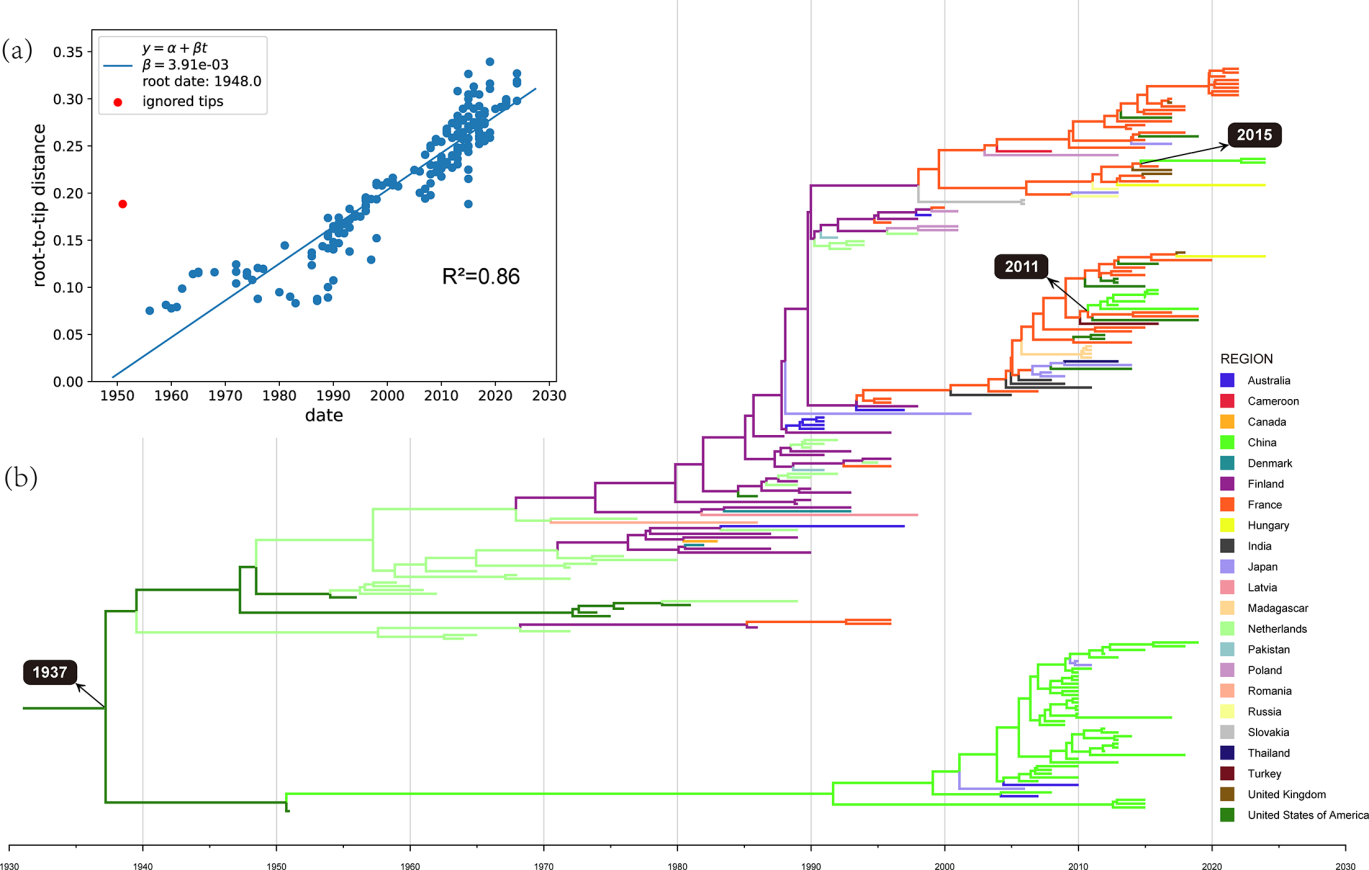
(**a**) The outcomes of root-to-tip regression analysis, where *β* represents the inferred molecular clock rate. (**b**) The MCC tree based on the dataset CVB4 reference VP1 (Table S4). The tMRCA was estimated to be 1937, while the tMRCA of the two Chinese CVB4 genotype D lineages was 2011 and 2015, respectively.

Root-to-tip regression analysis assessing the temporal signal of the reference CVB4 VP1 sequences yielded a correlation coefficient of 0.93 and an *R*-squared value of 0.86, supporting the applicability of the sequence data for temporal phylogenetic analysis. Bayesian phylogenetic analysis estimated the time to the most recent common ancestor (tMRCA) of CVB4 to be ~1928 (Fig. 4). Since then, CVB4 has diverged into two major global lineages. Lineage I consisted predominantly (95%) of Chinese strains, including genotype E (the dominant genotype in China) and genotype A. Genotype A included the prototype CVB4 strain (X05690, the USA, 1951) and three strains (OP342797, OP342799 and OP342802) detected in 2015 in Yunnan Province, China. Lineage II included genotypes B, C and D, mainly distributed in Europe. Genotypes B and C were last reported in Australia (GU142875, 1997) and France (AF160001-AF160006, 1996), respectively, after which only genotype D continued to be detected. Genotype D became globally widespread with a European centre, suggesting cross-border transmission. In this study, Z168 and Z296 might indicate an emerging cross-border transmission in recent years.

**Fig. 5.**
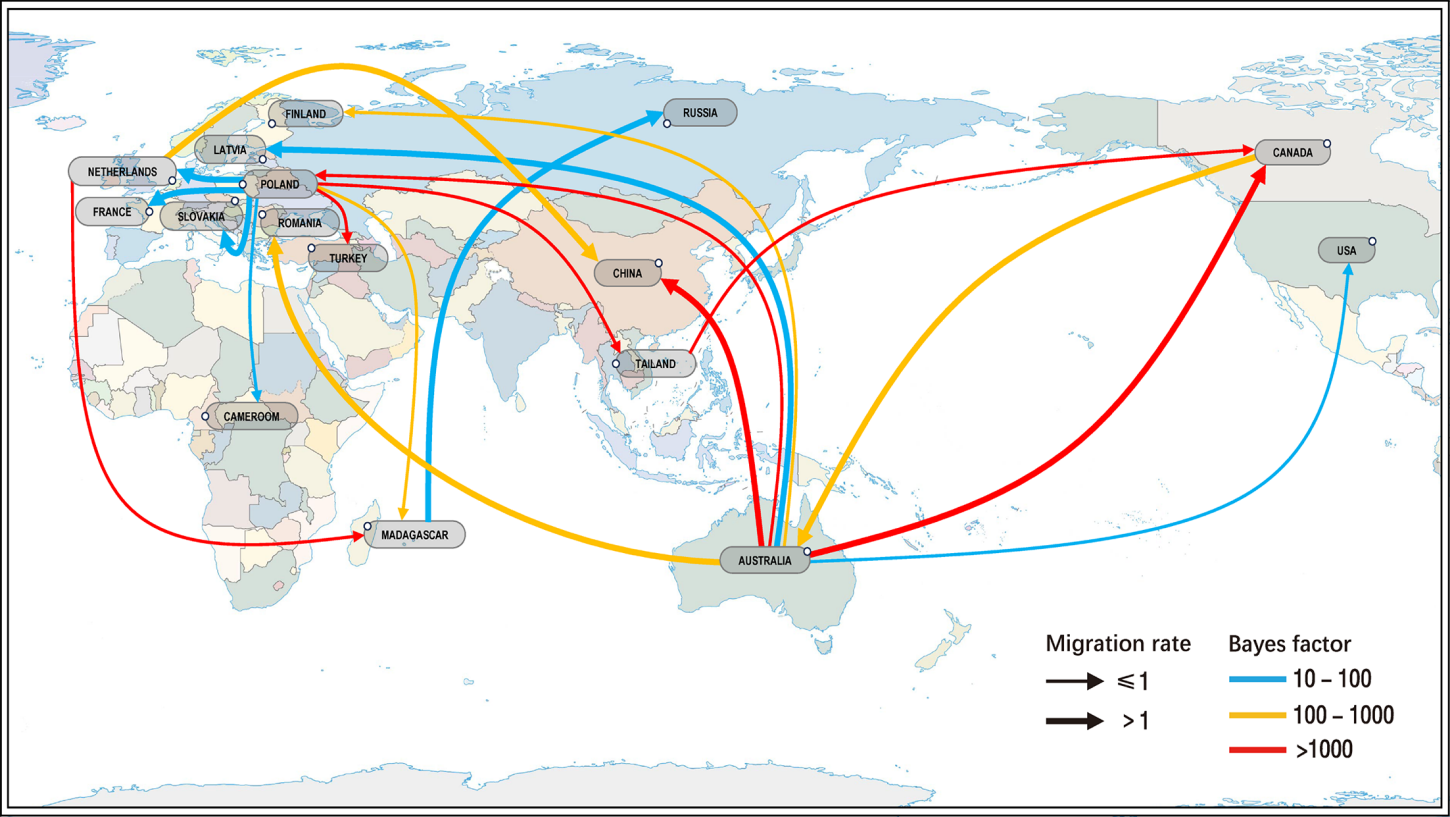
Spatial transmission routes of CVB4 inferred through Bayesian phylogeographic analysis based on the dataset CVB4 reference VP1 (Table S4). The thickness of the lines corresponds to the migration rate, and the colour indicates the robustness based on BF values.

Moreover, phylogeographic analyses were conducted to reconstruct historical transmission routes and characterize spatial transmission patterns (Fig. 5). The BaTS indicated significant geographic clustering of CVB4 (Table S9). Based on the MCC tree and node state posterior probabilities, the initial origin of CVB4 was inferred to be the USA. Between 1950 and 1990, genotype A spread from the USA to China; genotype E, evolved from genotype A, originated in China since 1990; and genotype D originated in the Netherlands since 1969. In 2011, CVB4 genotype D was introduced from France into China and subsequently circulated there for the first time, with strains from Yunnan (*n*=26; 2013–2016) and Tianjin (MZ161144, 2019) belonging to this cluster. Between 2014 and 2021, another cluster of genotype D was introduced from France to China and circulated independently; the two genotype D strains identified in this study belonged to this latter cluster. Further phylogeographic analysis using the BSSVS method revealed 20 statistically significant migration routes based on BF and PP thresholds (Table S10). A spatial transmission network highlighted Australia and Poland as major hubs of global CVB4 dissemination, each acting as the origin for 7 of the 20 significant transmission routes. Frequent bidirectional gene flow was observed within Europe and between Europe and Australia. Among the supported routes, the highest transmission rate was observed from Poland to France and the Netherlands to China, with a rate of 1.091.

### Amino acid substitution

A total of 15 amino acid substitution sites were identified in Z168 and Z296, in comparison with consensus sequences of genotypes A, D and E strains isolated in China (Table 2).

**Table 2.** Amino acid substitutions between Z168, Z296 and the consensus sequences of Chinese Coxsackievirus B4 genotypes A, D and E

Site		3	5	6	11	64	71	263	271	273	274	277	279	281	283	284
Strain																
Genotype A consensus sequence		T	E	S	M	I	S	N	V	A	E	S	I	T	P	Y
Genotype D consensus sequence		T	E	S	M	I	S	N	I	T	K	S	V	T	P	C
Genotype E consensus sequence		T	E	A	M	V	S	N	V	A	E	S	I	T	L	Q
Z168 (genotype D)		A	D	S	I	I	A	N	V	N	E	N	I	T	P	R
Z296 (genotype D)		T	D	S	I	I	A	S	V	T	E	N	I	N	P	R

**Fig. 6.**
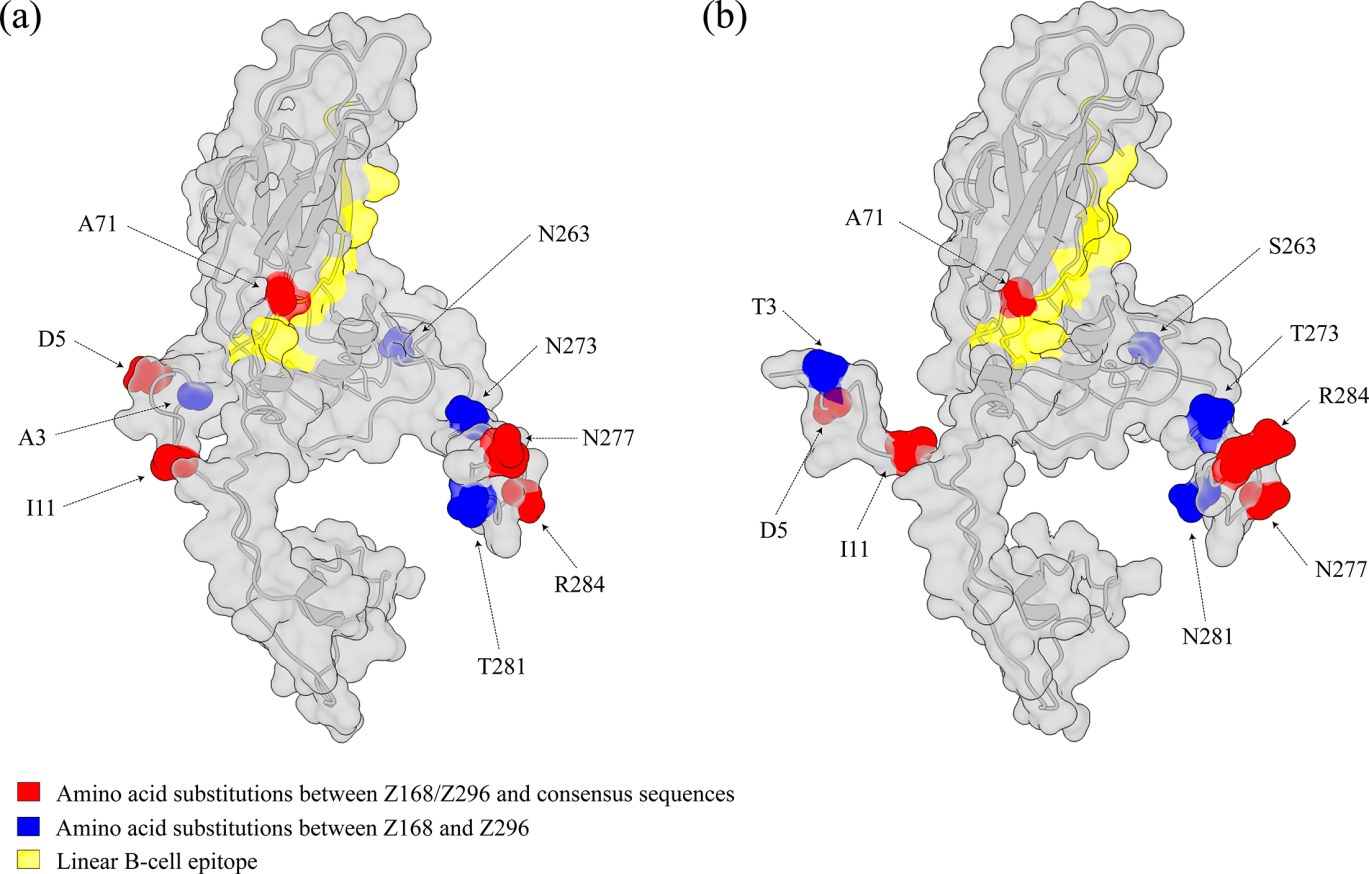
Secondary structure of CVB4 VP1 sequences from (**a**) Z168 and (**b**) Z296 strains isolated in this study. Amino acid substitutions are highlighted, in comparison with Chinese CVB4 VP1 amino acid consensus sequences.

Among these substitution positions, four sites differentiated Z168 from Z296, whereas five sites were identical in Z168 and Z296 but differed from the genotype A/D/E consensus sequences, and these substitutions were mainly located near the N- and C-terminal regions of the VP1 polypeptide. Notably, amino acid substitution at site 71 was located within a linear B-cell epitope spanning residues 68 to 82 of VP1, which had been identified as a potential serotype-specific neutralizing antigenic site [36]. At this site, all three consensus strains from China had a serine residue, whereas both Z168 and Z296 had an alanine residue. Using protein modelling of the VP1 region, amino acid substitutions within Z168 and Z296 strains were visualized to better understand their structural context (Fig. 6).

## Discussion

In this study, through the herpangina sentinel surveillance conducted in 2024 in Yuhuan City, Zhejiang Province, eastern China, we identified and sequenced two CVB4 strains, Z168 and Z296, and further classified them as genotype D that is a genotype rarely reported in China. In contrast, genotype E was determined to be the predominant CVB4 genotype in China. Moreover, we characterized the molecular and phylogenetic features of these two CVB4 strains, indicating a cross-border transmission to Yuhuan City, an island in the coastal area in eastern China. By integrating recently published data, we updated the current understanding of the spatiotemporal dynamics of CVB4, thereby complementing findings from previous research.

CVB4 is globally widespread [13]. Our results show that Z168 and Z296 exhibited significantly higher nucleotide and amino acid similarity with other CVB4 strains isolated in multiple countries. Notably, recombination analysis indicated that Z168 showed a recombination event involving a major parental CVB4 strain from France (2015) and a minor parental E11 strain from Guangdong Province, China (2019); similarly, Z296 was likely a recombinant between a major parental CVB4 strain from France (2017) and a minor parental CVB5 strain from Hubei Province, China (2019). Given the high frequency of recombination among enteroviruses, it is reasonable to infer that the geographic origin of the parental strains may be more informative than serotype in tracing the transmission history [37,39]. It is thus plausible to hypothesize that the two CVB4 genotype D strains were closely related to those French strains that have been continuously circulating and contributing to recombination with other enteroviruses. Moreover, phylogenetic analysis revealed that the two CVB4 genotype D strains might be introduced into China from France between 2014 and 2021. Two previous reports from China had also identified genotype D strains, which constituted a Chinese genotype D lineage. However, in our study, the two CVB4 genotype D strains were phylogenetically distant from the above lineage, suggesting a novel CVB4 genotype D lineage in China. Thus, our study might indicate a cross-border transmission event in recent years, which might be attributable to human mobilization as Yuhuan City is a typical international commerce hub in eastern China.

Based on the present findings, it may be necessary to further current understanding of the spatiotemporal dynamics of CVB4. Previous studies concluded that a dominant genotype replacement event occurred in the 1990s, during which genotype D completely replaced the previously predominant genotypes B and C in Europe. Since then, only genotypes D (widespread prevalence across the world) and E (geographically restricted distribution in China) have circulated, resulting in a pattern of geographically independent circulation [13]. However, our study calls for a reconsideration of this view. Despite the significant geographic clustering of CVB4 suggested by BaTS, cross-border transmission has occurred throughout nearly 70 years of CVB4 circulation [9]. Notably, in the past two decades, ongoing transmission events between China and multiple countries have been repeatedly observed [14,17], including the two genotype D strains identified in this study that likely originated from France before introduction into China. In the context of globalization, there is little reason to believe that the geographically independent circulation pattern among CVB4 genotypes could be sustained. Instead, a shift in the global distribution pattern of CVB4 may be underway, and the potential public health implications of this transition require close monitoring. In particular, close tracking of confirmed key transmission events – such as the emergence and circulation of the novel genotype D lineage identified in this study – is essential for timely intervention and effective prevention of potential future outbreaks.

Several studies indicate that specific amino acid substitutions can modulate CVB4 virulence. In experimental models, a threonine at VP1 position 129 has been identified as a key determinant of virulence and antigenicity, and substitutions at this residue (e.g. Thr129→Met) have been shown to alter disease severity and B-cell recognition [36]. In our study, we identified a novel genotype D lineage in China that shares a unique amino acid substitution at position 71 of the VP1 protein. Although specific investigations of the association between this site and CVB4 virulence are currently lacking, there is evidence that this residue is located within a well-defined, serotype-specific neutralizing epitope. A substitution from a polar to a non-polar amino acid within this key antigenic epitope could substantially alter local conformation and surface properties, potentially affecting neutralizing antibody recognition and binding [40]. It has also been demonstrated in experimental studies of other enteroviruses [41,42]. Further studies are therefore needed to clarify the potential role of this lineage-specific VP1-71 substitution in CVB4 virulence and antigenicity.

Enhanced large-scale, both cross-sectional and longitudinal, surveillance of CVB4 is urgently and critically needed. Current monitoring of CVB4 is severely inadequate, with a substantial proportion of infections remaining undetected [13], which likely biases our understanding of its global distribution patterns. A typical example has been documented that CVB4 genotype A strains were isolated in Yunnan Province, China, in 2015 [14], challenging the prevailing notion that only genotypes D and E circulated following the dominant genotype replacement event [13]. This finding suggests that genotype A was not completely displaced after its introduction into China and subsequent evolution into genotype D but rather has likely circulated undetected at low levels for decades. It aligns with previous perspectives indicating that CVB4 has long been, and continues to be, substantially under-monitored [9,13]. Additionally, our study revealed that the novel genotype D lineage shared a unique amino acid substitution at site 71 of the VP1 protein, located within a well-defined serotype-specific neutralizing [36], potentially affecting neutralizing antibody recognition and binding. Our findings likely reflect adaptive evolution of CVB4 following natural transmission or immune pressure, indicating the emergence of antigenically distinct viral subpopulations and underscoring the importance of enhanced and sustained virological surveillance.

There were several study limitations to the study. First, the surveillance was geographically restricted to a single sentinel site in Yuhuan City, Zhejiang Province, which may not fully represent the diversity and distribution of CVB4 across China. Second, phylogeographic and recombination analyses provided insights into the possible origins and transmission routes; however, the conclusion was conducted based on currently available sequences in the public database, which might remain incomplete and subject to regional and temporal biases. Third, amino acid substitutions were identified in key antigenic regions; in contrast, functional validation through serological assays was not performed, and thus, the possible impact on antigenicity or immune evasion remains speculative. Finally, the limited sample size prevented assessment of the potential association between viral genetic variation and disease severity.

In conclusion, our study identified two CVB4 genotype D strains in eastern China. They were phylogenetically closed to those European genotype D strains (isolated between 2013 and 2024), whereas distant from previously reported genotype D strains in China (between 2013 and 2019). Our findings further indicated cross-border transmission and subsequent adaptive evolution. Thus, sentinel surveillance may facilitate characterization of CVB4 and other enterovirus transmission.

## Supplementary material

10.1099/mgen.0.001656 Uncited Supplementary Material 1.

## Abbreviations

AI: association index
BF: Bayes factor
BSSVS: Bayesian Stochastic Search Variable Selection
CDS: coding sequence
CT: cycle threshold
CVB4: Coxsackievirus B4
CVBs: group B Coxsackieviruses
HFMD: hand, foot and mouth disease
MCC: maximum clade credibility
MCMC: Markov Chain Monte Carlo
NJ: neighbour-joining
PP: posterior probability
tMRCA: time to the most recent common ancestor
