# Supplementary material for: Full-genome analysis of emerging Coxsackievirus B4 genotype D strains associated with herpangina in eastern China
Source: Microb Genom. 2026 Feb 26;12(2):001656. doi: 10.1099/mgen.0.001656 (PMC12943721; doi:10.1099/mgen.0.001656)
Supplement: Uncited Supplementary Material 1. [file mgen-12-01656-s001.pdf]

Table S1. Collection information in the dataset Coxsackievirus B4 CDS.

Accession	Collection country	Collection year
AF311939	-	-
DQ480420	Italy	-
JX308222	China	2010
JX417724	Cameroon	2008
KC558559	Denmark	-
KC558560	Denmark	-
KC558561	Denmark	-
KC558562	Denmark	-
KC558563	Denmark	-
KC558564	Denmark	-
KC558565	Denmark	-
KC558566	Denmark	-
KC558567	Denmark	-
KC558568	Denmark	-
KC558569	Denmark	-
KC558570	Denmark	-
KC558571	Denmark	-
KC558572	Denmark	-
KC558573	Denmark	-
KF781524	China	2010
KF781525	China	2010
KM890276	China	2009
KP289433	China	2013
KU566507	China	2013
KY369904	The United State	2016
LS451289	Romania	1986
MF179585	China	2013
MF422559	Taiwan, China	2008
MF422560	Taiwan, China	2008
MF422562	Taiwan, China	2008
MF678300	Australia	2010
MF678319	Australia	2010
MF678347	Australia	2007
MG451808	The United Kingdom	2017
MG845888	The United State	2015
MG845889	The United State	2017
MN590273	France	2019
MW015036	Japan	2006
MW015037	Japan	2008
MW015038	Japan	2009
MW015039	Japan	2010

Accession	Collection country	Collection year
MW015040	Japan	2011
MW015041	Japan	2013
MW015042	Japan	2014
MW015043	Japan	2017
MW015044	Japan	2002
MW390784	Slovakia	2006
MW390785	Slovakia	2006
MW390786	Slovakia	2006
MW390787	Slovakia	2006
MW390788	Slovakia	2006
MW390789	Slovakia	2006
MZ161144	China	2019
MZ540957	China	2015
MZ540958	China	2018
MZ540959	China	2018
MZ540960	China	2018
OM417124	Thailand	2013
OQ791553	The United State	2014
OQ842446	The United State	2012
OQ842447	The United State	2012
OQ842448	The United State	2012
OQ842450	The United State	2013
OQ842451	The United State	2013
PP558442	France	2022
PP558443	France	2022
PP558444	France	2021
PP558445	France	2021
PP558446	France	2022
PP558447	France	2022
PP558448	France	2022
PP558449	France	2022
PP558450	France	2022
PP558451	France	2022
PP558452	France	2022
PP558453	France	2022
PP558454	France	2022
PP558455	France	2022
PP558456	France	2022
PP558457	France	2022
PP558458	France	2017
PP558459	France	2017
PP558460	France	2018
PP558461	France	2014

Accession	Collection country	Collection year
PP558462	France	2015
PP558463	France	2017
PP558464	France	2018
PP558465	France	2018
PP558466	France	2015
PP558467	France	2015
PP558468	France	2016
PP558469	France	2016
PP558470	France	2016
PP558471	France	2015
PP558472	France	2016
PP558473	France	2015
PP558474	France	2015
PP558475	France	2014
PP558476	France	2015
PP558478	France	2017
PP558479	France	2014
PP558480	France	2014
PP558481	France	2015
PQ248920	Hungary	2024
PQ335016	Hungary	2024
PQ350321	China	2015
S76772	The United State	1956
X05690	The United State	1951
Z168	China	2024
Z296	China	2024

Table S2. Collection information in the dataset Coxsackievirus B4+CVB-like CDS.

Accession	Serotype	Collection country	Collection year
D00627	Coxsackievirus A9	The United State	1950
M16560	Coxsackievirus B1	Japan	1987
AF081485	Coxsackievirus B2	The United State	1947
M88483	Coxsackievirus B3	The United State	1949
JX417724	Coxsackievirus B4	Cameroon	2008
KC558559	Coxsackievirus B4	Denmark	2013
KF781525	Coxsackievirus B4	China	2010
KM890276	Coxsackievirus B4	China	2009
LS451289	Coxsackievirus B4	Romania	1986
MG845889	Coxsackievirus B4	The United State	2017
MW015041	Coxsackievirus B4	Japan	2013
MW015044	Coxsackievirus B4	Japan	2002
MW390784	Coxsackievirus B4	Slovakia	2006
MZ161144	Coxsackievirus B4	China	2019
MZ540959	Coxsackievirus B4	China	2018
PP558451	Coxsackievirus B4	France	2022
PQ335016	Coxsackievirus B4	Hungary	2024
PQ350321	Coxsackievirus B4	China	2015
X05690	Coxsackievirus B4	The United State	1951
Z168	Coxsackievirus B4	China	2024
Z296	Coxsackievirus B4	China	2024
AF114383	Coxsackievirus B5	The United State	1952
AF039205	Coxsackievirus B6	Philippines	1953
AF029859	Echovirus 1	Egypt	1951
X80059	Echovirus 11	The United State	1953
AY302539	Echovirus 13	Philippines	1953
AY302542	Echovirus 16	The United State	1951
AY302546	Echovirus 20	The United State	1955
AY302548	Echovirus 24	The United State	1956
AY302550	Echovirus 26	Philippines	1953
AF162711	Echovirus 30	The United State	1958
AY302557	Echovirus 4	The United State	1951
AY302558	Echovirus 6	The United State	1955
AY302560	Enterovirus B69	Mexico	1959

Table S3. Collection information in the dataset Coxsackievirus B4 VP1.

Accession	Collection country	Collection year
AF159998	Netherlands	1965
AF159999	Netherlands	1964
AF160000	Netherlands	1972
AF160001	France	1996
AF160002	France	1996
AF160003	France	1996
AF160004	France	1996
AF160005	France	1996
AF160006	France	1996
AF160007	Finland	1986
AF160008	Netherlands	1977
AF160009	Romania	1986
AF160010	Denmark	1993
AF160011	Denmark	1993
AF160012	Finland	1993
AF160013	Latvia	1998
AF160014	Finland	1990
AF160015	Finland	1989
AF160016	France	1996
AF160017	France	1996
AF160018	Pakistan	1992
AF160019	Pakistan	1992
AF160020	Netherlands	1993
AF160021	Netherlands	1993
AF160022	Netherlands	1994
AF160023	Netherlands	1994
AF160024	France	1996
AF160025	Finland	1998
AF160026	Finland	1998
AF160027	Netherlands	1998
AF160028	Finland	1996
AF160029	Netherlands	1992
AF160030	Netherlands	1990
AF160031	Finland	1989
AF160032	Netherlands	1989
AF160033	The United State	1986
AF160034	Finland	1993
AF160035	Finland	1990
AF160036	Netherlands	1995
AF160037	Netherlands	1995
AF160038	Finland	1996

Accession	Collection country	Collection year
AF160039	France	1996
AF160040	Finland	1988
AF160041	Finland	1988
AF160042	Netherlands	1992
AF160043	Netherlands	1990
AF160044	Netherlands	1991
AF160045	Finland	1991
AF160046	Finland	1993
AF160047	Pakistan	1991
AF160048	Netherlands	1976
AF160049	Netherlands	1980
AF160050	Netherlands	1974
AF160051	Netherlands	1972
AF160052	Netherlands	1968
AF160053	Netherlands	1972
AF160054	Netherlands	1965
AF160055	Netherlands	1989
AF160056	Netherlands	1989
AF160057	Netherlands	1989
AF160058	Finland	1989
AF160059	Finland	1989
AF160060	Finland	1987
AF160061	Canada	1983
AF160062	Denmark	1982
AF160063	Finland	1987
AF160064	Finland	1990
AF160065	Netherlands	1960
AF160066	Netherlands	1959
AF160067	Netherlands	1961
AF160068	Netherlands	1962
AF160069	The United State	1981
AF160070	The United State	1976
AF160071	The United State	1974
AF160072	The United State	1975
AF160073	Netherlands	1989
FJ868288	Australia	1991
FJ868289	Australia	1991
FJ868330	Australia	1991
FJ868331	Australia	1991
FJ868332	Australia	1991
GU142875	Australia	1997
GU142876	Australia	1997
GU142877	Australia	1999

Accession	Collection country	Collection year
HF948089	France	2000
HF948090	France	2007
JF794741	China	2010
JN016524	China	2010
JX308222	China	2010
JX417724	Cameroon	2008
JX513577	India	2009
JX513579	India	2009
JX513580	India	2009
JX513581	India	2009
JX513583	India	2008
KC867091	China	2008
KC867092	China	2008
KC867093	China	2009
KF177118	India	2011
KF412921	India	2005
KF781519	China	2007
KF781520	China	2010
KF781521	China	2010
KF781522	China	2010
KF781523	China	2010
KF781524	China	2010
KF781525	China	2010
KJ933328	China	2013
KJ933329	China	2013
KM890276	China	2009
KP289433	China	2013
KR232695	Madagascar	2011
KR232711	Madagascar	2011
KR232712	Madagascar	2011
KR232730	Madagascar	2011
KR232731	Madagascar	2011
KR232732	Madagascar	2011
KR232739	Madagascar	2011
KR232743	Madagascar	2011
KR232751	Madagascar	2011
KR232758	Madagascar	2011
KR232760	Madagascar	2011
KR232761	Madagascar	2011
KR232763	Madagascar	2011
KR232775	Madagascar	2011
KR232776	Madagascar	2011
KR232778	Madagascar	2011

Accession	Collection country	Collection year
KR232782	Madagascar	2011
KR232792	Madagascar	2011
KR232793	Madagascar	2011
KR232796	Madagascar	2011
KU189236	Poland	2001
KU189240	Poland	2001
KU189242	Poland	2001
KU189251	Poland	2013
KU566507	China	2013
KU841463	Russia	2013
KU841464	Russia	2013
KX752784	China	2013
KY369904	The United State	2016
LC412980	China	2017
LS451289	Romania	1986
MF179585	China	2013
MF179586	China	2014
MF179587	China	2013
MF179588	China	2013
MF422558	China	2008
MF422559	China	2008
MF422560	China	2008
MF422561	China	2008
MF422562	China	2008
MF422563	China	2008
MF678300	Australia	2010
MF678319	Australia	2010
MF678347	Australia	2007
MG451808	The United Kingdom	2017
MG845888	The United State	2015
MG845889	The United State	2017
MG922519	China	2012
MG922520	China	2012
MG922521	China	2012
MG922522	China	2012
MG922523	China	2012
MK044541	Turkey	2016
MN018196	China	2010
MN541060	China	2018
MN590273	France	2019
MN896919	The United State	2019
MN896920	The United State	2019
MN896921	The United State	2019

Accession	Collection country	Collection year
MT109013	China	2010
MT109014	China	2010
MT109015	China	2010
MT109016	China	2010
MT109017	China	2010
MT109018	China	2010
MT109019	China	2010
MT109020	China	2010
MT109021	China	2010
MT109022	China	2010
MT109023	China	2010
MT109024	China	2010
MT109025	China	2010
MT109026	China	2010
MT109027	China	2011
MT109028	China	2011
MT109029	China	2011
MT109030	China	2011
MT109031	China	2011
MT109032	China	2011
MT109033	China	2011
MT109034	China	2011
MT109035	China	2011
MT109036	China	2011
MT109037	China	2011
MT109038	China	2011
MT109039	China	2011
MT109040	China	2011
MT109041	China	2011
MT109042	China	2011
MT109043	China	2011
MT109044	China	2011
MT109045	China	2011
MT109046	China	2011
MT109047	China	2011
MT641353	The United Kingdom	2017
MT641357	The United Kingdom	2017
MT641411	The United Kingdom	2018
MW015036	Japan	2006
MW015037	Japan	2008
MW015038	Japan	2009
MW015039	Japan	2010
MW015040	Japan	2011

Accession	Collection country	Collection year
MW015041	Japan	2013
MW015042	Japan	2014
MW015043	Japan	2017
MW015044	Japan	2002
MW179456	China	2019
MW390784	Slovakia	2006
MW390785	Slovakia	2006
MW390786	Slovakia	2006
MW390787	Slovakia	2006
MW390788	Slovakia	2006
MW390789	Slovakia	2006
MZ161144	China	2019
MZ540957	China	2015
MZ540958	China	2018
MZ540959	China	2018
MZ540960	China	2018
OM417124	Thailand	2013
OP342795	China	2013
OP342796	China	2013
OP342797	China	2015
OP342798	China	2015
OP342799	China	2015
OP342800	China	2015
OP342801	China	2015
OP342802	China	2015
OP342803	China	2015
OP342804	China	2015
OP342805	China	2015
OP342806	China	2015
OP342807	China	2016
OP342808	China	2016
OP342810	China	2016
OP342812	China	2016
OP342813	China	2016
OP342814	China	2016
OP342815	China	2016
OP342816	China	2016
OP342817	China	2016
OP342818	China	2016
OP342819	China	2016
OP342820	China	2016
OP342821	China	2016
OP342822	China	2016

Accession	Collection country	Collection year
OP342825	China	2016
OP342826	China	2016
OP342827	China	2016
OP376489	China	2011
OP376490	China	2011
OP376491	China	2011
OP376492	China	2011
OP376493	China	2011
OP376494	China	2011
OP376495	China	2011
OP376496	China	2011
OP376497	China	2011
OP376498	China	2011
OP376499	China	2011
OP376500	China	2011
OP376501	China	2011
OP376502	China	2011
OP376503	China	2011
OP376504	China	2011
OP376505	China	2011
OP376506	China	2011
OP376507	China	2011
OP376508	China	2011
OP376509	China	2011
OP376510	China	2011
OP376511	China	2010
OP376512	China	2010
OP376513	China	2010
OP376514	China	2010
OP376515	China	2010
OP376516	China	2010
OP376517	China	2010
OP376518	China	2010
OP376519	China	2010
OP376520	China	2010
OP376521	China	2010
OP376522	China	2010
OP376523	China	2010
OP376524	China	2010
OP376525	China	2010
OP376526	China	2010
OP376527	China	2010
OP376528	China	2010

Accession	Collection country	Collection year
OQ791553	The United State	2014
OQ842446	The United State	2012
OQ842447	The United State	2012
OQ842448	The United State	2012
OQ842450	The United State	2013
OQ842451	The United State	2013
PP558442	France	2022
PP558443	France	2022
PP558444	France	2021
PP558445	France	2021
PP558446	France	2022
PP558447	France	2022
PP558448	France	2022
PP558449	France	2022
PP558450	France	2022
PP558451	France	2022
PP558452	France	2022
PP558453	France	2022
PP558454	France	2022
PP558455	France	2022
PP558456	France	2022
PP558457	France	2022
PP558458	France	2017
PP558459	France	2017
PP558460	France	2018
PP558461	France	2014
PP558462	France	2015
PP558463	France	2017
PP558464	France	2018
PP558465	France	2018
PP558466	France	2015
PP558467	France	2015
PP558468	France	2016
PP558469	France	2016
PP558470	France	2016
PP558471	France	2015
PP558472	France	2016
PP558473	France	2015
PP558474	France	2015
PP558475	France	2014
PP558476	France	2015
PP558477	France	2020
PP558478	France	2017

Accession	Collection country	Collection year
PP558479	France	2014
PP558480	France	2014
PP558481	France	2015
PQ248920	Hungary	2024
PQ335016	Hungary	2024
PQ350321	China	2015
S76772	The United State	1956
X05690	The United State	1951
Z168	China	2024
Z296	China	2024

Table S4. Collection information in the dataset Coxsackievirus B4 reference VP1.

Accession	Collection country	Collection year
AF159998	Netherlands	1965
AF159999	Netherlands	1964
AF160000	Netherlands	1972
AF160001	France	1996
AF160003	France	1996
AF160007	Finland	1986
AF160008	Netherlands	1977
AF160009	Romania	1986
AF160010	Denmark	1993
AF160012	Finland	1993
AF160013	Latvia	1998
AF160014	Finland	1990
AF160015	Finland	1989
AF160016	France	1996
AF160017	France	1996
AF160018	Pakistan	1992
AF160020	Netherlands	1993
AF160022	Netherlands	1994
AF160023	Netherlands	1994
AF160024	France	1996
AF160025	Finland	1998
AF160026	Finland	1998
AF160027	Netherlands	1998
AF160028	Finland	1996
AF160029	Netherlands	1992
AF160030	Netherlands	1990
AF160031	Finland	1989
AF160032	Netherlands	1989
AF160033	The United State	1986
AF160034	Finland	1993
AF160035	Finland	1990
AF160036	Netherlands	1995
AF160038	Finland	1996
AF160039	France	1996
AF160040	Finland	1988
AF160042	Netherlands	1992
AF160043	Netherlands	1990
AF160044	Netherlands	1991
AF160045	Finland	1991
AF160046	Finland	1993
AF160047	Pakistan	1991

Accession	Collection country	Collection year
AF160048	Netherlands	1976
AF160049	Netherlands	1980
AF160050	Netherlands	1974
AF160051	Netherlands	1972
AF160052	Netherlands	1968
AF160053	Netherlands	1972
AF160054	Netherlands	1965
AF160055	Netherlands	1989
AF160058	Finland	1989
AF160060	Finland	1987
AF160061	Canada	1983
AF160062	Denmark	1982
AF160063	Finland	1987
AF160064	Finland	1990
AF160065	Netherlands	1960
AF160066	Netherlands	1959
AF160067	Netherlands	1961
AF160068	Netherlands	1962
AF160069	The United State	1981
AF160070	The United State	1976
AF160071	The United State	1974
AF160072	The United State	1975
AF160073	Netherlands	1989
FJ868288	Australia	1991
FJ868289	Australia	1991
FJ868330	Australia	1991
FJ868332	Australia	1991
GU142875	Australia	1997
GU142876	Australia	1997
GU142877	Australia	1999
HF948089	France	2000
HF948090	France	2007
JF794741	China	2010
JX308222	China	2010
JX417724	Cameroon	2008
JX513577	India	2009
JX513583	India	2008
KC867091	China	2008
KC867092	China	2008
KC867093	China	2009
KF177118	India	2011
KF412921	India	2005
KF781519	China	2007

Accession	Collection country	Collection year
KF781520	China	2010
KF781521	China	2010
KF781522	China	2010
KF781525	China	2010
KJ933328	China	2013
KM890276	China	2009
KP289433	China	2013
KR232695	Madagascar	2011
KR232711	Madagascar	2011
KR232712	Madagascar	2011
KR232775	Madagascar	2011
KU189236	Poland	2001
KU189240	Poland	2001
KU189242	Poland	2001
KU189251	Poland	2013
KU566507	China	2013
KU841463	Russia	2013
KU841464	Russia	2013
KX752784	China	2013
KY369904	The United State	2016
LC412980	China	2017
MF179585	China	2013
MF179586	China	2014
MF179587	China	2013
MF422558	China	2008
MF678300	Australia	2010
MF678347	Australia	2007
MG451808	The United Kingdom	2017
MG845888	The United State	2015
MG845889	The United State	2017
MG922519	China	2012
MG922520	China	2012
MG922523	China	2012
MK044541	Turkey	2016
MN018196	China	2010
MN541060	China	2018
MN590273	France	2019
MN896919	The United State	2019
MN896921	The United State	2019
MT109013	China	2010
MT109014	China	2010
MT109015	China	2010
MT109016	China	2010

Accession	Collection country	Collection year
MT109020	China	2010
MT109027	China	2011
MT641353	The United Kingdom	2017
MT641357	The United Kingdom	2017
MT641411	The United Kingdom	2018
MW015036	Japan	2006
MW015037	Japan	2008
MW015038	Japan	2009
MW015039	Japan	2010
MW015040	Japan	2011
MW015041	Japan	2013
MW015042	Japan	2014
MW015043	Japan	2017
MW015044	Japan	2002
MW179456	China	2019
MW390784	Slovakia	2006
MW390786	Slovakia	2006
MZ161144	China	2019
MZ540957	China	2015
MZ540958	China	2018
OM417124	Thailand	2013
OP342795	China	2013
OP342797	China	2015
OP342798	China	2015
OP342799	China	2015
OP342800	China	2015
OP342802	China	2015
OP342807	China	2016
OP342810	China	2016
OP376511	China	2010
OP376512	China	2010
OP376528	China	2010
OQ791553	The United State	2014
OQ842446	The United State	2012
OQ842448	The United State	2012
OQ842450	The United State	2013
OQ842451	The United State	2013
PP558442	France	2022
PP558443	France	2022
PP558444	France	2021
PP558449	France	2022
PP558450	France	2022
PP558452	France	2022

Accession	Collection country	Collection year
PP558455	France	2022
PP558457	France	2022
PP558458	France	2017
PP558459	France	2017
PP558460	France	2018
PP558461	France	2014
PP558462	France	2015
PP558463	France	2017
PP558464	France	2018
PP558465	France	2018
PP558466	France	2015
PP558467	France	2015
PP558468	France	2016
PP558471	France	2015
PP558472	France	2016
PP558473	France	2015
PP558475	France	2014
PP558476	France	2015
PP558477	France	2020
PP558478	France	2017
PP558479	France	2014
PP558480	France	2014
PP558481	France	2015
PQ248920	Hungary	2024
PQ335016	Hungary	2024
S76772	The United State	1956
X05690	The United State	1951
Z168	China	2024
Z296	China	2024

Table S5. Collection information in the dataset Z168 BLAST CDS.

Accession	Serotype	Collection country	Collection year
KC897073	Echovirus 30	China	2012
KF781524	Coxsackievirus B4	China	2010
KM890276	Coxsackievirus B4	China	2009
KP289433	Coxsackievirus B4	China	2013
KP289438	Coxsackievirus B5	China	2013
KP289441	Echovirus 14	China	2013
KU574625	Coxsackievirus B5	Thailand	2011
KU566507	Coxsackievirus B4	China	2013
KX752784	Coxsackievirus B4	China	2013
KY369904	Coxsackievirus B4	The United State	2016
KX171337	Enterovirus B106	China	2011
MF422558	Coxsackievirus B4	China	2008
MF422560	Coxsackievirus B4	China	2008
MF422561	Coxsackievirus B4	China	2008
MF678300	Coxsackievirus B4	Australia	2010
MF678347	Coxsackievirus B4	Australia	2007
MG451808	Coxsackievirus B4	The United Kingdom	2017
MG845888	Coxsackievirus B4	The United State	2015
MG845889	Coxsackievirus B4	The United State	2017
MF179585	Coxsackievirus B4	China	2013
MF179587	Coxsackievirus B4	China	2013
MH144607	Enterovirus B88	India	2012
LS451289	Coxsackievirus B4	Romania	1986
MN145871	Echovirus 6	China	2017
MN580136	Enterovirus B93	China	1999
MK791151	Echovirus 6	China	2018
MN597925	Echovirus 11	China	2019
MN597928	Echovirus 11	China	2019
MN597930	Echovirus 11	China	2019
MN597931	Echovirus 11	China	2019
MN597932	Echovirus 11	China	2019
MN597933	Echovirus 11	China	2019
MN597937	Echovirus 11	China	2018
MN597940	Echovirus 11	China	2019
MN597942	Echovirus 11	China	2019
MN597943	Echovirus 11	China	2018
MN597944	Echovirus 11	China	2019
MN597945	Echovirus 11	China	2019
MN597946	Echovirus 11	China	2019
MN597947	Echovirus 11	China	2019
MN597949	Echovirus 11	China	2019

Accession	Serotype	Collection country	Collection year
MN597951	Echovirus 11	China	2019
MN597953	Echovirus 11	China	2019
MN597954	Echovirus 11	China	2019
MW015036	Coxsackievirus B4	Japan	2006
MW015037	Coxsackievirus B4	Japan	2008
MW015038	Coxsackievirus B4	Japan	2009
MW015039	Coxsackievirus B4	Japan	2010
MW015040	Coxsackievirus B4	Japan	2011
MW015041	Coxsackievirus B4	Japan	2013
MW015042	Coxsackievirus B4	Japan	2014
MW015044	Coxsackievirus B4	Japan	2002
MW883611	Echovirus 11	China	2019
MW883612	Echovirus 11	China	2019
MW883614	Echovirus 11	China	2019
MW390784	Coxsackievirus B4	Slovakia	2006
MW390785	Coxsackievirus B4	Slovakia	2006
MW390786	Coxsackievirus B4	Slovakia	2006
OL519575	Echovirus 11	China	2018
JX417724	Coxsackievirus B4	Cameroon	2008
OK632348	Coxsackievirus B3	China	2016
MW365443	Coxsackievirus B2	China	2019
MZ161144	Coxsackievirus B4	China	2019
MZ229642	Echovirus 11	China	2019
MZ229645	Echovirus 11	China	2019
MZ229648	Echovirus 11	China	2019
MZ229652	Echovirus 11	China	2019
MZ229655	Echovirus 11	China	2019
MZ229657	Coxsackievirus B5	China	2019
MZ540958	Coxsackievirus B4	China	2018
MZ540957	Coxsackievirus B4	China	2015
MW481633	Echovirus 16	China	2010
OP672304	Coxsackievirus B5	China	2016
OP376489	Coxsackievirus B4	China	2011
OP376511	Coxsackievirus B4	China	2010
OP376512	Coxsackievirus B4	China	2010
OP376514	Coxsackievirus B4	China	2010
OP376515	Coxsackievirus B4	China	2010
OP376528	Coxsackievirus B4	China	2010
OQ791553	Coxsackievirus B4	The United State	2014
OQ842446	Coxsackievirus B4	The United State	2012
OQ842448	Coxsackievirus B4	The United State	2012
OQ969172	Echovirus 11	France	2022
MZ488277	Echovirus 9	China	2019

Accession	Serotype	Collection country	Collection year
OR039178	Echovirus 11	China	2019
PP265499	Echovirus 11	China	2019
PP265500	Echovirus 11	China	2019
PP265502	Echovirus 11	China	2021
PP558442	Coxsackievirus B4	France	2022
PP558443	Coxsackievirus B4	France	2022
PP558447	Coxsackievirus B4	France	2022
PP558449	Coxsackievirus B4	France	2022
PP558451	Coxsackievirus B4	France	2022
PP558452	Coxsackievirus B4	France	2022
PP558454	Coxsackievirus B4	France	2022
PP558455	Coxsackievirus B4	France	2022
PP558456	Coxsackievirus B4	France	2022
PP558457	Coxsackievirus B4	France	2022
PP558458	Coxsackievirus B4	France	2017
PP558459	Coxsackievirus B4	France	2017
PP558460	Coxsackievirus B4	France	2018
PP558461	Coxsackievirus B4	France	2014
PP558462	Coxsackievirus B4	France	2015
PP558463	Coxsackievirus B4	France	2017
PP558464	Coxsackievirus B4	France	2018
PP558465	Coxsackievirus B4	France	2018
PP558466	Coxsackievirus B4	France	2015
PP558467	Coxsackievirus B4	France	2015
PP558470	Coxsackievirus B4	France	2016
PP558471	Coxsackievirus B4	France	2015
PP558472	Coxsackievirus B4	France	2016
PP558473	Coxsackievirus B4	France	2015
PP558475	Coxsackievirus B4	France	2014
PP558476	Coxsackievirus B4	France	2015
PP558477	Coxsackievirus B4	France	2020
PP558478	Coxsackievirus B4	France	2017
PP558479	Coxsackievirus B4	France	2014
PP558480	Coxsackievirus B4	France	2014
PP558481	Coxsackievirus B4	France	2015
PP558491	Echovirus 11	France	2022
PQ350320	Coxsackievirus B3	China	2014
PQ248920	Coxsackievirus B4	Hungary	2024
PQ335016	Coxsackievirus B4	Hungary	2024
PQ572233	Enterovirus B88	Russia	2023
PP552734	Echovirus 11	Spain	2023
PP552736	Echovirus 11	Spain	2022
X05690	Coxsackievirus B4	The United State	1951

Accession	Serotype	Collection country	Collection year
JX308222	Coxsackievirus B4	China	2010
KF781525	Coxsackievirus B4	China	2010
MN590273	Coxsackievirus B4	France	2019
MW015043	Coxsackievirus B4	Japan	2017
OM417124	Coxsackievirus B4	Thailand	2013
OQ842450	Coxsackievirus B4	The United State	2013
Z168	Coxsackievirus B4	China	2024

Table S6. Collection information in the dataset Z296 BLAST CDS.

Accession	Serotype	Collection country	Collection year
KF312882	Enterovirus B111	China	2000
KP289438	Coxsackievirus B5	China	2013
JX476169	Coxsackievirus B3	India	2009
KY369904	Coxsackievirus B4	The United State	2016
KX171337	Enterovirus B106	China	2011
MG451808	Coxsackievirus B4	The United Kingdom	2017
LC191400	Coxsackievirus B2	Japan	2008
MG845888	Coxsackievirus B4	The United State	2015
MN145871	Echovirus 6	China	2017
MK791151	Echovirus 6	China	2018
MN597925	Echovirus 11	China	2019
MN597928	Echovirus 11	China	2019
MN597930	Echovirus 11	China	2019
MN597931	Echovirus 11	China	2019
MN597932	Echovirus 11	China	2019
MN597933	Echovirus 11	China	2019
MN597937	Echovirus 11	China	2018
MN597940	Echovirus 11	China	2019
MN597942	Echovirus 11	China	2019
MN597943	Echovirus 11	China	2018
MN597944	Echovirus 11	China	2019
MN597945	Echovirus 11	China	2019
MN597946	Echovirus 11	China	2019
MN597947	Echovirus 11	China	2019
MN597949	Echovirus 11	China	2019
MN597951	Echovirus 11	China	2019
MN597953	Echovirus 11	China	2019
MN597954	Echovirus 11	China	2019
MN590273	Coxsackievirus B4	France	2019
MW015037	Coxsackievirus B4	Japan	2008
MW015038	Coxsackievirus B4	Japan	2009
MW015041	Coxsackievirus B4	Japan	2013
MW015042	Coxsackievirus B4	Japan	2014
MW015043	Coxsackievirus B4	Japan	2017
MW015044	Coxsackievirus B4	Japan	2002
MW883611	Echovirus 11	China	2019
MW883612	Echovirus 11	China	2019
MW883614	Echovirus 11	China	2019
MW390785	Coxsackievirus B4	Slovakia	2006
MW390786	Coxsackievirus B4	Slovakia	2006
OL519575	Echovirus 11	China	2018

Accession	Serotype	Collection country	Collection year
JX417724	Coxsackievirus B4	Cameroon	2008
OK632344	Coxsackievirus B3	China	2016
MZ161144	Coxsackievirus B4	China	2019
MZ229645	Echovirus 11	China	2019
MZ229648	Echovirus 11	China	2019
MZ229652	Echovirus 11	China	2019
MZ229655	Echovirus 11	China	2019
MZ229657	Coxsackievirus B5	China	2019
OM417124	Coxsackievirus B4	Thailand	2013
OP672304	Coxsackievirus B5	China	2016
OQ791553	Coxsackievirus B4	The United State	2014
OQ791570	Echovirus 31	The United State	2016
OQ842446	Coxsackievirus B4	The United State	2012
OQ842448	Coxsackievirus B4	The United State	2012
OQ842450	Coxsackievirus B4	The United State	2013
OQ969172	Echovirus 11	France	2022
OR039175	Echovirus 11	China	2019
OR039177	Echovirus 11	China	2019
OR039178	Echovirus 11	China	2019
PP265499	Echovirus 11	China	2019
PP265500	Echovirus 11	China	2019
PP265502	Echovirus 11	China	2021
PP558442	Coxsackievirus B4	France	2022
PP558443	Coxsackievirus B4	France	2022
PP558447	Coxsackievirus B4	France	2022
PP558449	Coxsackievirus B4	France	2022
PP558451	Coxsackievirus B4	France	2022
PP558452	Coxsackievirus B4	France	2022
PP558454	Coxsackievirus B4	France	2022
PP558455	Coxsackievirus B4	France	2022
PP558456	Coxsackievirus B4	France	2022
PP558457	Coxsackievirus B4	France	2022
PP558458	Coxsackievirus B4	France	2017
PP558459	Coxsackievirus B4	France	2017
PP558460	Coxsackievirus B4	France	2018
PP558461	Coxsackievirus B4	France	2014
PP558462	Coxsackievirus B4	France	2015
PP558463	Coxsackievirus B4	France	2017
PP558464	Coxsackievirus B4	France	2018
PP558465	Coxsackievirus B4	France	2018
PP558466	Coxsackievirus B4	France	2015
PP558467	Coxsackievirus B4	France	2015
PP558470	Coxsackievirus B4	France	2016

Accession	Serotype	Collection country	Collection year
PP558471	Coxsackievirus B4	France	2015
PP558472	Coxsackievirus B4	France	2016
PP558473	Coxsackievirus B4	France	2015
PP558475	Coxsackievirus B4	France	2014
PP558476	Coxsackievirus B4	France	2015
PP558478	Coxsackievirus B4	France	2017
PP558479	Coxsackievirus B4	France	2014
PP558480	Coxsackievirus B4	France	2014
PP558481	Coxsackievirus B4	France	2015
PP558491	Echovirus 11	France	2022
PQ350320	Coxsackievirus B3	China	2014
PQ248920	Coxsackievirus B4	Hungary	2024
PQ335016	Coxsackievirus B4	Hungary	2024
PQ572233	Enterovirus B88	Russia	2023
PP552734	Echovirus 11	Spain	2023
PP552736	Echovirus 11	Spain	2022
JN797614	Echovirus 30	Switzerland	2006
JN797615	Coxsackievirus B1	Switzerland	2010
JN797616	Echovirus 30	Switzerland	2009
JX308222	Coxsackievirus B4	China	2010
KF781525	Coxsackievirus B4	China	2010
KP289433	Coxsackievirus B4	China	2013
KX752784	Coxsackievirus B4	China	2013
MF422560	Coxsackievirus B4	China	2008
MF422561	Coxsackievirus B4	China	2008
MG845889	Coxsackievirus B4	The United State	2017
MH144607	Enterovirus B88	India	2012
LS451289	Coxsackievirus B4	Romania	1986
MN166092	Echovirus 18	The United State	2015
MW365443	Coxsackievirus B2	China	2019
MZ229642	Echovirus 11	China	2019
Z296	Coxsackievirus B4	China	2024

Table S7. Marginal likelihood estimation of the molecular clock model and coalescent model in the dataset Coxsackievirus B4 reference VP1.

Molecular clock model	Coalescent tree prior	PS[a]	SS[b]
Strict clock	GMRF Bayesian Skyride	-23225.2	-23216.5
Strict clock	Constant size	-22950	-22950.1
Strict clock	Exponential growth	-22950.3	-22950.6
Strict clock	Bayesian Skyline	-22947.9	-22947.1
Strict clock	Expansion	-22952.4	-22953
Strict clock	SkyGrid	-23094.6	-23090.2
Uncorrelated exponential relaxed clock	GMRF Bayesian Skyride	-23003.4	-22997.1
Uncorrelated exponential relaxed clock	Constant size	-22800.7	-22802.2
Uncorrelated exponential relaxed clock	Exponential growth	-22792	-22792.8
Uncorrelated exponential relaxed clock	Bayesian Skyline	-22793.5	-22795.3
**Uncorrelated exponential relaxed clock**	**Expansion**	**-22608.4**	**-22612.6**
Uncorrelated exponential relaxed clock	SkyGrid	-22808.8	-22809.5
Uncorrelated lognormal relaxed clock	GMRF Bayesian Skyride	-22841.8	-22843.2
Uncorrelated lognormal relaxed clock	Constant size	-22815.4	-22816.2
Uncorrelated lognormal relaxed clock	Exponential growth	-22809.4	-22809
Uncorrelated lognormal relaxed clock	Bayesian Skyline	-22821.5	-22824
Uncorrelated lognormal relaxed clock	Expansion	-23052	-23045.5
Uncorrelated lognormal relaxed clock	SkyGrid	-23007.9	-22996.3

The best-fitting model combination was highlighted in bolded font.

[a] PS, Path sampling

[b] SS, Stepping-stone sampling

Table S8. Recombination results obtained by software RDP4 analysis.

	PARENT	Accession	Serotype	Genotype	Collection year	Collection country	Source	Tissue Specimen Source	Patient's information
Z168	MAJOR	PP558466	Coxsackievirus B4	D	2015	France	Human	throat swab	8-year-old boy
	MINOR	MN597951	Echovirus 11	-	2019	Guangdong, China	Sewage	-	-
Z296	MAJOR	PP558458	Coxsackievirus B4	D	2017	France	Human	throat swab	18-month-old child with fever and herpangina
	MINOR	MZ229657	Coxsackievirus B5	-	2019	Hubei, China	Human	throat swab	-

Table S9. Bayesian Tip-Significance Testing for spatial structure of Coxsackievirus B4 in the dataset Coxsackievirus B4 reference VP1.

Statistic	observed mean (95% HPD CIs)[d]	null mean (95% HPD CIs)	P-value
AI[a]	7.4 (6.8,8.0)	18.9 (17.8,19.9)	<0.001
PS[b]	69.8 (68.0,71.0)	133.6 (128.8,139.4)	<0.001
MC (the Netherlands)[c]	3.8 (3.0,4.0)	1.8 (1.1,3.0)	0.0
MC (France)	8.2 (8.0,10.0)	2.1 (1.7,3.0)	0.0
MC (Finland)	2.0 (2.0,2.0)	1.3 (1.0,2.0)	0.1
MC (Romania)	1.0 (1.0,1.0)	1.0 (1.0,1.0)	1.0
MC (Denmark)	1.0 (1.0,1.0)	1.0 (1.0,1.0)	1.0
MC (Latvia)	1.0 (1.0,1.0)	1.0 (1.0,1.0)	1.0
MC (Pakistan)	1.0 (1.0,1.0)	1.0 (1.0,1.0)	1.0
MC (the United States)	2.0 (2.0,2.0)	1.4 (1.0,2.0)	0.2
MC (Canada)	1.0 (1.0,1.0)	1.0 (1.0,1.0)	1.0
MC (Australia)	4.0 (4.0,4.0)	1.1 (1.0,1.8)	0.0
MC (China)	10.7 (10.0,14.0)	2.3 (1.9,3.1)	0.0
MC (Cameroon)	1.0 (1.0,1.0)	1.0 (1.0,1.0)	1.0
MC (India)	1.0 (1.0,1.0)	1.0 (1.0,1.0)	1.0
MC (Madagascar)	4.0 (4.0,4.0)	1.0 (1.0,1.0)	0.0
MC (Poland)	2.0 (2.0,2.0)	1.0 (1.0,1.0)	0.0
MC (Russia)	1.0 (1.0,1.0)	1.0 (1.0,1.0)	1.0
MC (the United Kingdom)	1.0 (1.0,1.0)	1.0 (1.0,1.0)	1.0
MC (Turkey)	1.0 (1.0,1.0)	1.0 (1.0,1.0)	1.0
MC (Japan)	2.0 (2.0,2.0)	1.1 (1.0,1.8)	0.0
MC (Slovakia)	2.0 (2.0,2.0)	1.0 (1.0,1.0)	0.0
MC (Thailand)	1.0 (1.0,1.0)	1.0 (1.0,1.0)	1.0
MC (Hungary)	1.0 (1.0,1.0)	1.0 (1.0,1.0)	1.0

[a] AI, association index.

[b] PS, parsimony score.

[c] MC, maximum monophyletic clade.

[d] HPD CIs, highest posterior density confidence intervals.

Table S10. Statistically significant transmission routes based on BF and PP thresholds.

FROM	TO	Mean migration rate	BF[a]	PP[b]
Netherlands	Madagascar	0.920	2206.9	0.991
Australia	Poland	0.949	11221.1	0.998
Australia	Canada	1.011	6930.8	0.997
Australia	Romania	1.039	597.1	0.967
Australia	Latvia	1.014	30.2	0.598
Poland	France	1.091	20.5	0.502
Poland	Slovakia	1.037	56.5	0.736
Poland	Madagascar	0.963	161.4	0.888
Poland	Cameroon	0.949	38.6	0.656
Poland	Turkey	0.917	18267343.3	1.000
Poland	Thailand	0.896	405921.1	1.000
Madagascar	Russia	1.059	22.0	0.520
Netherlands	China	1.091	274.6	0.931
Australia	China	1.079	1724.8	0.988
Australia	The United State	0.971	26.6	0.567
Poland	Netherlands	1.066	35.9	0.639
Australia	Finland	0.939	135.8	0.870
Canada	Australia	1.033	574.2	0.966
Thailand	Canada	0.888	1442.7	0.986

[a] BF, Bayes factor.

[b] PP, Posterior Probability.

Table S11. Primers used for enterovirus typing

Primer	Sequence (5′–3′)
EV2	ATTGTCACTGGATGGCCAAT
EV4	CAAGCACTTCTGTTTCCCCGG

Table S12. Primers for CVB4 whole-genome amplification

Primer pair	Forward primer (5′–3′)	Reverse primer (5′–3′)
CVB4_5F / CVB4_1589R	AACAGCCTGTGGGTTGTWC	CATYTTGTCCATTGGYACACTG
CVB4_1458F / CVB4_2922R	GTGTGCAATGCAGGTATGG	GACATTCTTGGTGGTGCATTGC
CVB4_2693F / CVB4_4391R	TGARTCAAACAACTTGAAGC	GCATTTGGACTTGAACTGTA
CVB4_4239F / CVB4_5915R	ACCATCCCAGAGTGACCAG	ATCTCACCYTGCTCATCRTT
CVB4_5706F / CVB4_7392R	TGGTCAAGTGACAGATTATG	ACCGAATGCGGAGAATTTAC

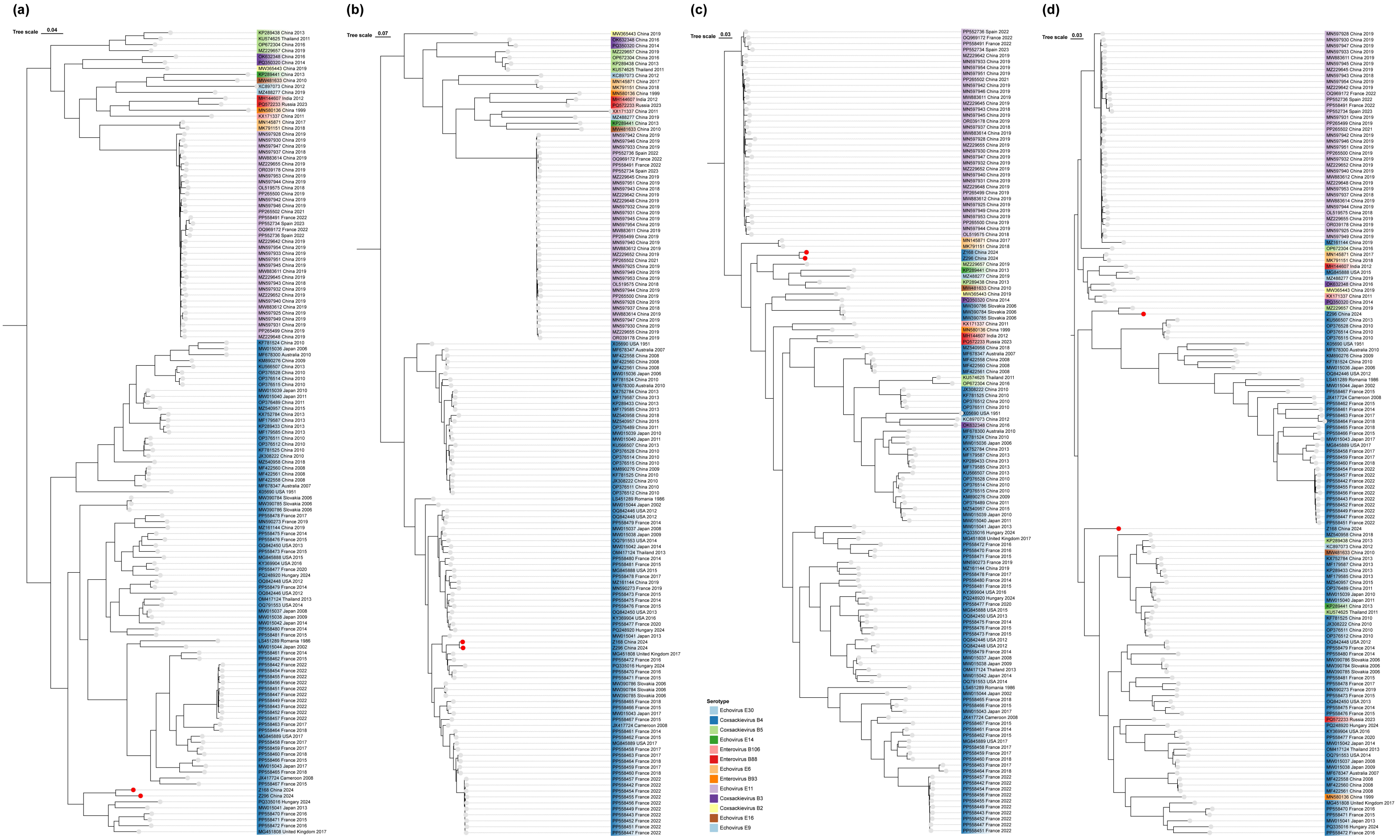

Figure S1. Maximum-likelihood (ML) trees reconstructed from the full CDS, P1, P2, and P3 regions of the Z168 BLAST dataset (Table S5), comprising CVB4 sequences and their potential recombinant counterparts. Panels corresponded to analyses based on (a) the full CDS, (b) P1, (c) P2, and (d) P3 regions, respectively. The red dots represented the strains isolated in this study.

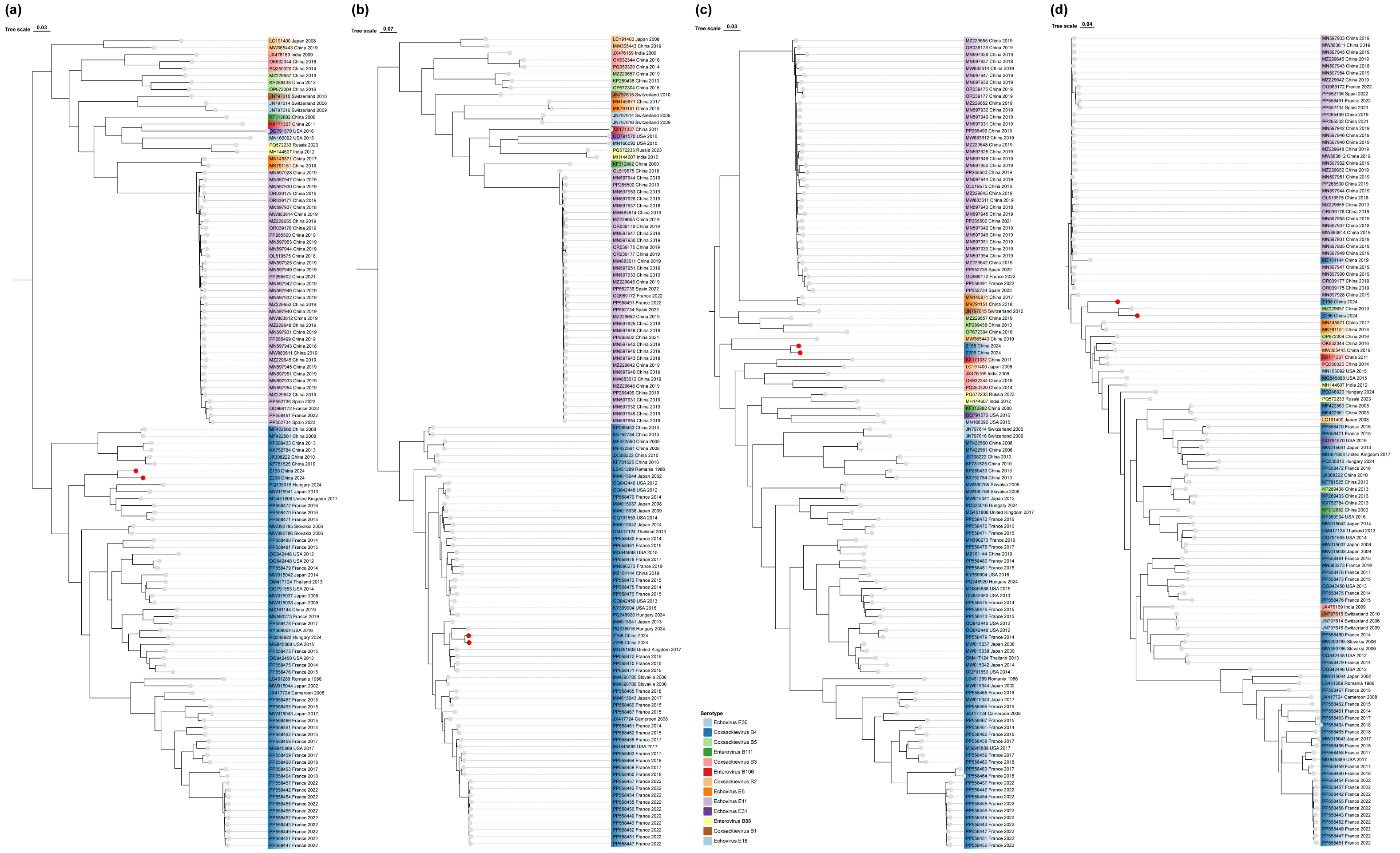

Figure S2. Maximum-likelihood (ML) trees reconstructed from the full CDS, P1, P2, and P3 regions of the Z296 BLAST dataset (Table S5), comprising CVB4 sequences and their potential recombinant counterparts. Panels corresponded to analyses based on (a) the full CDS, (b) P1, (c) P2, and (d) P3 regions, respectively. The red dots represented the strains isolated in this study.
